# Flat-Back vs. Arched-Back Bench Press: Examining the Different Techniques Performed by Power Athletes

**DOI:** 10.1519/JSC.0000000000004778

**Published:** 2024-03-29

**Authors:** Sandro Bartolomei, Emanuele Caroli, Vittorio Coloretti, Giuseppe Rosaci, Matteo Cortesi, Giuseppe Coratella

**Affiliations:** 1 Department for Life Quality Studies, Università di Bologna, Rimini, Italy; and; 2 Department of Biomedical Sciences for Health, Università degli Studi di Milano, Milan, Italy

**Keywords:** resistance training, powerlifting, strength, velocity-based training, electromyography, pectoralis major

## Abstract

Bartolomei, S, Caroli, E, Coloretti, V, Rosaci, G, Cortesi, M, and Coratella, G. Flat-back vs. arched-back bench press: Examining the different techniques performed by power athletes. *J Strength Cond Res* 38(7): 1200–1205, 2024—The International Powerlifting Federation recently changed the regulations concerning the bench press (BP) technique, not allowing an accentuated dorsal arch anymore. We investigated the difference between the flat-back vs. arched-back BP performed by competitive powerlifters as concerns the following parameters: (a) 1 repetition maximum (1RM) and barbell displacement; (b) mean and peak barbell velocity and power, and (c) the excitation of the prime movers. Fifteen highly resistance trained individuals (BP 1RM/body mass ratio: 1.38 ± 0.18) performed the flat-back and arched-back BP at their 50, 70, and 90% of the respective 1RM and performed each lift with the intent to maximally accelerate the barbell. Barbell displacement and velocity, power, and the excitation of the upper and lower pectoralis and triceps brachii were assessed. The 1RM was greater with the arched-back BP (+4.2 Kg, 95% confidence intervals + 0.0/+8.4, effect size [ES]: 0.22), whereas the barbell displacement was greater with the flat-back BP for all loads (ES from 0.40 to 0.61). Greater mean (+0.052 m·s^−1^, 0.016/0.088, ES: 0.42) and peak barbell velocity (+0.068 m·s^−1^, +0.026/0.110, ES: 0.27) were observed in the flat-back BP, whereas power did not differ. The excitation of upper and lower pectoralis was similar, while an overall trend for an increased activation of triceps brachii was noted in the arched-back vs. flat-back BP. Interestingly, no between-load difference in the excitation of upper and lower pectoralis was observed (*p* > 0.05). Depending on the training purposes, both flat-back and arched-back BP may be used. The present outcomes may assist practitioners and competitive powerlifters to inform training session.

## Introduction

Bench press (BP) is widely used in resistance training to stimulate the upper-body muscles. Several studies have examined its technique and prime movers (1,4) or the long-term training adaptations following different protocols of BP training (3,20). With the intent to increase the load and vary the stimuli, practitioners often adopt different strategies, such as adapting the bench inclination (4), increasing or decreasing the handgrip stance (14), or accentuating the dorsal arch while performing BP (27).

Compared with the traditional BP technique described by the National Strength and Conditioning Association (9), powerlifters often adopt a dorsal arch because it decreases the barbell displacement thus increasing stability, favoring higher loads to be lifted (27). Thus, many powerlifters have been able to strongly emphasize the dorsal arch, consequently much reducing the barbell displacement and facilitating the increment in BP load during competitions. As such, the ability to accentuate the dorsal arch has become gradually a strong determinant of the overall performance. Therefore, the International Powerlifting Federation (IPF) has recently changed the regulations concerning the BP technique during competitions. Particularly, while the dorsal arch was allowed without any specification until 2022, starting from January 2023, the elbows must be below the shoulders at the lowest point of the trajectory (13). Thus, the dorsal arch is much limited, and the new technique requires a flatter dorsal setting.

The difference between the flat-back vs. the old arched-back BP has been investigated in the literature, although not systematically. For instance, a study used the Smith machine that does not replicate the training and competitions motor patterns (8), whereas another study investigated Paralympics technique (23). Although established in the practice, a direct comparison of the 1 repetition maximum (1RM) between the flat-back vs. arched-back BP has been not reported; however, other studies have shown smaller barbell displacement using the arched-back technique (8,22). Moreover, practitioners perform BP using certain tempo for each phase (5) or may perform the concentric phase with the intent to maximally accelerate the barbell, based on the velocity-based training method (7). The comparison between the flat-back and old arched-back technique as concerns mean and peak barbell velocity and power has been examined only in 2 studies, albeit one used the Smith machine (8) and the other the paralympic technique (23). Finally, varying the technique may affect the prime mover activity, especially upper and lower pectoralis and triceps brachii, examined so far only in 1 study (6).

Notwithstanding, the change of regulations by the IPF requires the new arched-back technique to be investigated because avoiding the extreme accentuation of the dorsal arch may reduce the differences between the flat-back and the new arched-back technique. Therefore, the aims of this study were to investigate the difference between the flat-vs. arched-back BP performed by competitive powerlifters as concerns the following parameters: (a) the 1RM and barbell displacement, (b) the mean and peak barbell velocity and power, and (c) the excitation of the prime movers. For a more comprehensive analysis, we used light-to-heavy loads for both techniques. The results may potentially assist practitioners in planning training sessions for developing strength and upper-body muscle size and to prepare powerlifting competitions according to the new regulations.

## Methods

### Experimental Approach to the Problem

The present investigation was designed as a within-subject, cross-sectional study. The sample size was calculating a priori using a statistical software (G*Power 3.1, University of Dusseldorf, Germany). Considering the study design, α level = 0.05, a desired power *β* = 0.80, nonsphericity correction = 1, correlation among repeated measures = 0.6, and effect size (ES) *f* = 0.25 (medium), the sample size resulted in 16 subjects. After recruiting the desired number of subjects, 1 had to dropout due to personal reasons.

The subjects reported to the laboratory on 3 separate occasions. During the first 2 visits, the subjects randomly performed the 1RM for the flat-back or arched-back BP. During the third visit, the subjects performed 1 set with 50, 70, and 90% 1RM for both the flat- and the arched-back BP, for a total of 6 sets. During each set, the mean and peak barbell velocity and power were recorded, together with the excitation of the upper and lower pectoralis major and triceps brachii. Each session was separated by at least 3 days. The subjects were instructed to avoid any form of heavy resistance training for the entire duration of the procedures.

### Subjects

Fifteen highly resistance trained people (13 men and 2 women) volunteered in this study (age: 29.0 ± 6.6 years; body mass: 87.5 ± 15.3 kg; height: 176.8 ± 8.0 cm). Ten subjects were highly resistance trained individuals with at least 3 years of experience and were familiar with both the flat-back and the arched-back BP. Ten of the subjects were competitive powerlifters, 3 were competitive weightlifters, and 2 were athletic throwers. The inclusion criteria required the subjects to be able to lift at least 1.3 times their body mass in the BP exercise using a FL technique (mean 1RM = 1.38 ± 0.18 body mass). The exclusion criteria included suffering any injury in the year before being enrollment in this study. The subjects were asked to abstain from alcohol, caffeine, and resistance training for at least 48 hours before all visits to laboratory. The testing procedures were fully explained before obtaining individual written informed consent after being informed about the risks and benefits of the study. The study was approved by the Ethical Committee of the University of Bologna University (n. 0025317; February 01, 2023).

### Procedures

*Bench Press Technique*: BP was performed using a horizontal bench (30-cm width, 45-cm height, 125-cm length; Technogym, Cesena, Italy). The flat-back technique was characterized by 5 points of contact: head, shoulder blades, thoracic trunk, buttocks, and feet, as described by the National Strength and Conditioning Association (9). This technique requires the subjects to maintain their natural lordosis of the lumbar spine, without any voluntary accentuation. The arched-back technique also includes 5 points of contact but requires an accentuation of the lumbar arch and retraction of the scapula (8). The new regulations require the elbow to be lowered below the shoulders at the lowest point of the trajectory (13). For both techniques, the barbell was lowered to the chest. The handgrip distance was free for each subject in accordance with their personal experience and comfort. This was measured and controlled in each set, and the subjects were asked to maintain the same handgrip distance when using the flat-back and the arched-back technique. The subjects were also required to maintain the heels on ground. The subjects were instructed to maximally accelerate the barbell during the ascending phase, whereas the descending phase was performed with a 2-s tempo provided by a computerized metronome. The subjects were required to maintain the aforementioned execution irrespective of the load.

### One Repetition Maximum

The 1RM procedures for both the flat-back and the arched-back BP were performed as previously described in the literature (11). Each subject was asked to complete 2 warm-up sets before starting with the 1RM test, using a resistance of 40–60% and 60–80% of his perceived maximum, respectively. After the specific warm-up, the subjects were required to perform a single repetition with each load that was incremented until failure. Each set was separated by 3 minutes of passive recovery. Standardized strong encouragements were provided by the operators at each attempt. Once the flat-back and the arched-back 1RM were determined, their 50, 70, and 90% were calculated for both techniques.

### Barbell Displacement, Velocity, and Power

The barbell displacement, velocity, and power during each lift at 50, 70, and 90% 1RM for both the flat-back and arched-back BP was recorded using an encoder (Tendo Unit model V104; Tendo Sports Machines, Trencin, Slovak Republic) and was performed on the same bench used for determining the 1RM. Each set consisted of 2 repetitions, and the order was randomized. A 3-minute recovery time was given between each set. The maximum barbell displacement was determined and inserted into the data analysis. In addition, the mean barbell velocity and power averaged across the 2 repetitions, and the peak barbell velocity and power recorded were calculated and inserted into the data analysis. All the measurements were collected during the ascending phase. The subjects were required to maintain the aforementioned execution irrespective of the load.

### Electromyographic Measurements

The electromyographic (EMG) signal was detected during the ascending phase of each repetition from the clavicular and sternocostal head of pectoralis major and the lateral head of triceps brachii. The skin area under the EMG electrodes was shaved, cleaned with ethyl alcohol, abraded gently with fine sand paper, and prepared with a conductive cream (Nuprep, Weaver and Co., Aurora, CO) to achieve an interelectrode impedance below 2000 Ω. EMG signal was detected by 2 Ag/AgCl rounded electrodes with solid hydro-gel (RAM apparecchi medicali s.r.l., Genova, Italy). Following the European Recommendations for Surface Electromyography (10), the electrodes were placed along the direction of the muscle fibers, between the tendon and the motor point. Particular care to the electrode placement was given because it was recently shown that the innervation zone of the pectoralis major shifts as a function of shoulder position in the BP (16). For example, if the electrode shifted over the innervation zone during part of the movement, the muscle excitation is underestimated. Therefore, to overcome such a possible bias, a fast Fourier transform approach was used, as suggested in a previous investigation (18). Briefly, the electrode placement on each muscle was checked during the warm-up phase of each exercise analyzing the power spectrum profile of the EMG signal recorded. The correct electrode placement results in a typical belly-shaped power spectrum profile of the EMG signal, while noise, motion artifacts, power line, electrodes placed on the innervation zone, or myotendinous junction generate a different power spectrum profile (18). The same experienced operator placed the electrodes and checked the power-spectrum profile.

The EMG electrodes for pectoralis major were placed on the midclavicular line, midway between the acromioclavicular joint of the shoulder for the clavicular head (26). For the sternocostal head, the electrodes were placed on the sternoclavicular joint of the sternum, over the second and fifth intercostals spaces (26). The sEMG electrode for the lateral head of triceps brachii was placed over the midbelly of the lateral head midway between the posterior crista of the acromion and the olecranon at 2-finger width lateral to the line (10).

The electrodes were equipped with a probe (probe mass: 8.5 g, BTS Inc., Milano, Italy) that permitted the detection and the transfer of the EMG signal by wireless modality. EMG signal was acquired at 1,000 Hz, amplified (gain: 2000, impedance and the common rejection mode ratio of the equipment are >10^15^ Ω//0.2 pF and 60/10 Hz 92 dB, respectively) and driven to a wireless electromyographic system (FREEEMG 1000, BTS Inc., Milano, Italy) that digitized (1,000 Hz) and filtered (band-pass 10–500 Hz) the raw EMG signals. The EMG signals from the ascending phase of each repetition were analyzed in time domain. In addition, a 25-millisecond mobile window was used for the computation of the root mean square (RMS). During each exercise, the RMS was calculated and averaged over the duration of the concentric phase and the 2 repetitions.

### Statistical Analyses

The statistical analysis was performed using a statistical software (SPSS 28.0.1.0, IBM, Armonk, NY). The Shapiro-Wilk test was used to assess the normal distribution of the data, and all dependent parameters resulted in a normal distribution (*p* > 0.05). If the assumption of sphericity was violated, a Greenhouse-Geisser correction was applied. The difference in 1RM and between the flat-back and arched-back BP was calculated using a T-test for pairwise groups. The differences in barbell displacement, mean and peak barbell velocity and power, and RMS of the clavicular and sternocostal head of pectoralis major and triceps brachii were calculated using a technique (2 levels: flat-back and arched-back BP) × load (3 levels: 50, 70, and 90% 1RM) repeated-measures analysis of variance. Multiple pairwise comparisons were performed using the Bonferroni's correction. Significance was set at *p* < 0.05. The ES was calculated for each pairwise comparison using the Cohen's *d* and was interpreted as follows: 0.00 to 0.19: trivial; 0.20 to 0.59: small; 0.60 to 1.19: moderate; 1.20 to 1.99: large; ≥2.00: very large (12). Descriptive statistics are reported as mean (*SD*). Significance was accepted at an alpha level of *p* ≤ 0.05.

## Results

**Figure 1.**
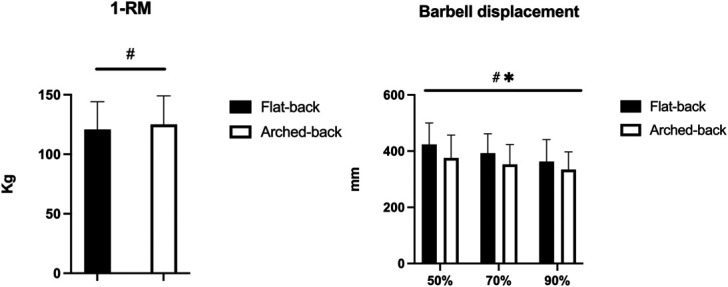
One repetition maximum and barbell displacement. #*p* < 0.05 between flat-back vs. arched-back. *p < 0.05 between the loads for both flat-back and arched-back.

The 1RM was greater using the arched-back vs. the flat-back BP (+4.2 Kg, 95% confidence intervals + 0.0 to +8.4, ES: 0.22, *p* = 0.031) (Figure 1).

No technique × load interaction was found for the barbell displacement (*p* = 0.277), while a main effect was observed for the technique factor (*p* < 0.001) and load (*p* < 0.001). Flat-back led to greater barbell displacement than arched-back at 50% (+47 mm, +34 to +61, ES: 0.61), 70% (+40 mm, +24 to +55, ES: 0.58), and 90% (+28 mm, +6 to +50, ES: 0.40) 1RM. For both the flat-back and the arched-back BP, the displacement decreased with incremental loads (*p* < 0.05 for all pairwise comparisons) (Figure 1).

**Figure 2.**
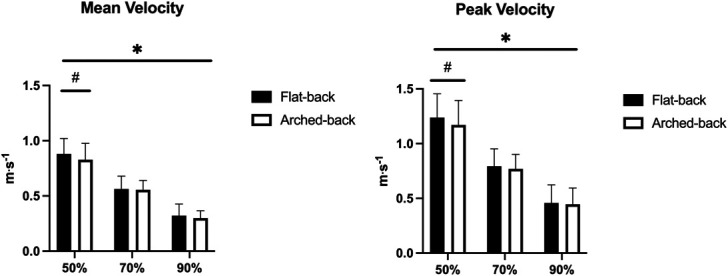
Mean and peak barbell velocity. #*p* < 0.05 between flat-back vs. arched-back. *p < 0.05 between the loads for both flat-back and arched-back.

No technique × load interaction was found for the mean (*p* = 0.306) and the peak barbell velocity (*p* = 0.146), while a main effect was observed for the technique factor (*p* = 0.017 and *p* = 0.037, respectively) and load (*p* < 0.001 for both the mean and the peak barbell velocity). Greater mean (+0.052 m·s^−1^, 0.016 to 0.088, ES: 0.42) and peak velocity (+0.068 m·s^−1^, +0.026 to 0.110, ES: 0.27) was found for the flat-back vs. the arched-back BP at 50% 1RM, while no further difference was observed. For both the flat-back and the arched-back BP, the mean and the peak barbell velocity decreased with incremental loads (*p* < 0.05 for all pairwise comparisons) (Figure 2).

**Figure 3.**
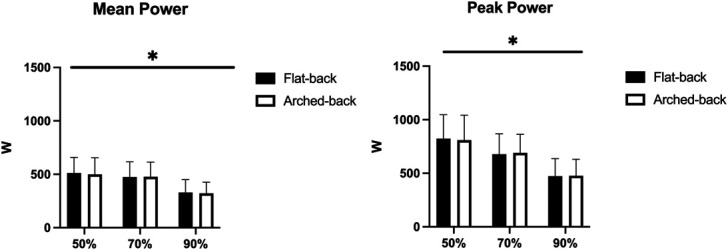
Mean and peak power. *p < 0.05 between the loads for both flat-back and arched-back.

No technique × load interaction was observed for the mean (*p* = 0.596) and the peak power (*p* = 0.532), nor main effect for the technique factor (*p* = 0.520 and 0.916, respectively), while a main effect was found for factor load (*p* < 0.001 for both the mean and the peak power). No difference in the mean and the peak power was observed comparing flat-back and arched-back BP. For both the flat-back and the arched-back BP, the mean and the peak power decreased with incremental loads (*p* < 0.05 for all pairwise comparisons) (Figure 3).

**Figure 4.**
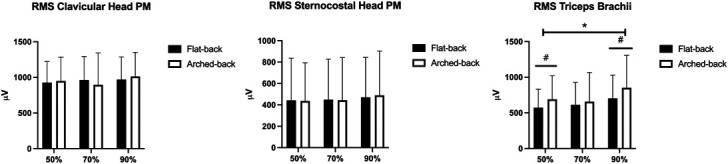
RMS of the clavicular and sternocostal head of pectoralis major and triceps brachii. PM = pectoralis major. #*p* < 0.05 between flat-back vs. arched-back. *p < 0.05 between 50 and 90% for both flat and arched back.

No technique × load interaction was found for the RMS of the clavicular (*p* = 0.495) and sternocostal (*p* = 0.693) head of pectoralis major or for main effect for the technique factor (*p* = 0.985 and *p* = 0.870, respectively) and load (*p* = 0.491 and *p* = 0.198). No between-exercise and between-load difference was observed in both muscles (Figure 4).

No technique × load interaction was found for the RMS of the triceps brachii (*p* = 0.364), or a main effect for the technique factor (*p* = 0.054) was observed. However, a trend for an increased excitation of the triceps brachii was observed in the arched-back technique compared with the flat-back technique. A main effect for factor load was registered (*p* = 0.017). The triceps brachii excitation was greater at 90 vs. 50% 1RM in both the arched-back (+161 μV, +27 to 296, ES: 0.40) and flat-back BP (+129 μV, +11 to +248, ES: 0.44). No further difference was observed (Figure 4).

## Discussion

This study was designed to investigate the differences between the flat-back vs. the new arched-back BP with different loads performed with the intent to maximally accelerate the barbell. We observed greater 1RM for the arched-back with lower displacement at all loads than the flat-back BP. In addition, while no between-exercise difference in mean and peak power occurred, the mean and peak barbell velocity were greater for the flat-back vs. arched-back BP at 50% 1RM. Concurrently, the increments in load led to decrements in both the mean and peak power and the mean and peak velocity for both the BP variations. Finally, no between-exercise and between-load difference in the excitation of the clavicular and sternocostal head of pectoralis major was observed. However, triceps brachii showed an overall trend for greater excitation for the arched-back vs. flat-back BP. Altogether, the present results suggest that the differences in flat-back vs. arched-back BP should be considered when training and preparing BP competitions.

We found greater 1RM for the arched-back vs. flat-back BP, although the difference was small. The most likely explanation for the greater 1RM using the new arched-back vs. flat-back BP is the smaller displacement, as also reported here for each load with a small-to-moderate magnitude. This allows the subjects to lift more weight, so that the arched-back technique is widely used during the powerlifting or BP competitions to improve performance (27). Incidentally, because many athletes emphasize the dorsal arch with a dramatic reduction in barbell displacement, this strategy has led to an increase in personal best performance. The arched-back technique may be even more effective in light body mass categories, with athletes able to perform a very pronounced dorsal arch to reduce the displacement and consequently the total work during each lift. The literature has already examined this comparison, and some authors observed similar results in experienced trained men (6) or paralympic athletes (23), while other authors did not find any difference in competitive male powerlifters (8). However, the latter study (8) was conducted using a Smith machine, and the guided trajectory of the barbell may have affected the results, given the greater stability of the whole movement. As concerns the barbell vertical displacement, previous studies showed similar results, with a reduction using the arched-back vs. flat-back BP using a wide range of loads in subjects with different training experience (17,19,22,23). Interestingly, the barbell displacement gradually decreased when incrementing the load. Although it may appear equivocal because one may expect that the start and the end of the movement should not change, the subjects were instructed to accelerate the barbell maximally. Consequently, lighter loads lead to increasing scapular protraction, thus increasing the final displacement. By contrast, heavier loads are more difficult to accelerate and do not consent to any further movement. This was visible for both the flat-back and the arched-back BP.

A small difference in mean and peak barbell velocity was found in favor of the flat-back vs. arched-back BP, although this was only visible at 50% 1RM. Because mean velocity is simply the barbell displacement divided by time, a between-technique difference may derive from the greater space the subjects had to accelerate the barbell. However, when incrementing the load this difference tended to disappear, possibly because the greater loads did not allow lifters to reach high barbell velocity. Previous studies did not find any between-technique difference in barbell velocity at various loads (8,23). Nonetheless, in 1 study, BP was performed on the Smith machine, so that the more stable trajectory could have helped to maintain similar linear barbell velocity (8), while the other study involved paralympic athletes who may have had a less pronounced arched-back technique compared with our subjects (23). As expected, incrementing the load led to diminishing the mean and peak barbell velocity in both the flat-back and the arched-back BP, which is in line with the force-velocity(max) principle (24).

Notwithstanding, the mean and peak power did not differ between the flat-back vs. arched-back BP. It should be noted that both the techniques were performed with the same relative loads, albeit the absolute loads differed given the greater 1RM achieved with the arched-back BP. Therefore, the subjects had to generate more force in the arched-back vs. flat-back BP, leading to a compensation in the overall power expressed. Our results are in line with the literature (8). Both the mean and peak power decreased when incrementing the load, in both the flat-back and arched-back BP. The literature has reported that the maximum power in BP is achieved with a load ranging from 30 to 60% 1RM (2,15), so it is likely that the present loads were in the descending portion of the power-load relationship for most subjects.

The present results did not show any between-technique and between-load difference in the excitation of both clavicular and sternocostal head of pectoralis. Comparing the flat-back vs. arched-back BP, another study did not find any difference in pectoralis excitation at light-to-heavy loads (6), so it appears that the dorsal position does not affect the excitation of the main chest muscles. Interestingly, the present outcomes showed that load did not affect the excitation of both heads of pectoralis major. Although theoretically the EMG amplitude is expected to be dependent on load (4,21), the intention to maximally accelerate the load may have mediated the excitation, possibly maximizing it at each load in both techniques.

Conversely, triceps brachii excitation tended to be greater (ES: small) in the arched-back vs. flat-back BP. A similar trend was also observed in the literature (6). In the arched-back BP, the barbell touches the chest at a lower elbow flexion, so the lifting phase starts with triceps brachii in a more favorable position to increment its activity albeit with a small advantage (6). In addition, it is possible that the arched-back BP had a more stable barbell trajectory, which in turn may increase the role of triceps brachii (28). Triceps brachii was also more excited at 90 vs. 50% 1RM in both flat-back and arched-back technique. It is possible that the load played a greater role than the intention to maximally accelerate the barbell as concerns triceps brachii (25).

Some limitations accompany this study. First, although the subjects were recruited on the basis of the previous 1RM and their powerlifting experience, it is possible that the personal lifting technique may not have been identical. However, we are confident of a certain consistency. Second, we did not collect data at 1RM. We acknowledge that this may have deepened the investigation, but the experimental design did not allow performing several maximal attempts without inducing fatigue. Thus, we opted for lighter loads. Third, we did not record any kinematic data that would be an interesting possibility for future studies. Finally, we limited the muscle excitation to 3 muscles, so that a more comprehensive analysis of the prime mover excitation should require more muscles like deltoids or latissimus dorsi.

In conclusion, this study analyzed the possible difference in the flat-back vs. arched-back BP. Greater 1RM but lower displacement was found in the arched-back BP, while the barbell velocity was higher in the flat-back BP only at light load, with no difference in power. The excitation of the upper and lower pectoralis did not differ, while triceps was overall more excited in the arched-back BP.

**Practical Applications**

The current outcomes could have some interesting impact on the practice of BP in a competitive setting. For example, the new arched-back BP allows greater load to be lifted, thus may be chosen when this is the main stimulus of the training cycle or session. By contrast, the flat-back BP favors higher barbell velocity to be reached, possibly aiming for improving explosive tasks. The excitation of the main chest muscles can be effectively stimulated with both techniques, while arched-back BP may enhance the excitation of the triceps brachii. In light of the present results, practitioners should not perform BP using the flat-back or arched-back technique exclusively but should be aware of the main differences and incorporate both techniques depending on the training objectives.
